# A case report of Covid-19 in an autoimmune pulmonary alveolar proteinosis: An association in tune with the times!

**DOI:** 10.1016/j.rmcr.2023.101825

**Published:** 2023-02-27

**Authors:** Valentin Coirier, Flora Delamaire, Pierre Chauvin, Mallorie Kerjouan, Mathieu Lederlin, Adel Maamar, Stéphane Jouneau

**Affiliations:** a Department of Infectious Diseases and Intensive Care, Rennes University Hospital, Rennes, France; b Department of Respiratory Diseases, Rennes University Hospital, Rennes, France; c Department of Radiology, Rennes University Hospital, Rennes, France; d IRSET UMR 1085, Rennes University, Rennes, France

## Abstract

Autoimmune pulmonary alveolar proteinosis (PAP) is a rare disease characterized by the alveoli accumulation of surfactants proteins and lipids, which diagnosis is confirmed by the presence of GM-CSF antibodies in serum. PAP can be evoked when its characteristic images on chest computed-tomography (CT) are present: bilateral and multifocal ground-glass opacities and crazy-paving appearance. Patients with PAP are at an increased risk of opportunistic infections caused by Nocardia, mycobacteria and fungal pathogens due to impaired processing of pulmonary surfactant. We here report a typical case of newly diagnosed autoimmune PAP, with initial indication to realize a whole-lung lavage. Despite this treatment the patient presented a marked clinical worsening, with increasing need for oxygen and finally the need for mechanical ventilation. The chest CT was controlled and found to be typical of PAP, while the search for opportunistic infections remained negative. Finally, SARS-CoV-2 PCR was performed on bronchoalveolar lavage fluid, and was positive, whereas it had previously been negative twice. Our case report highlights the difficulty of distinguishing SARS-CoV-2 infection in the context of PAP, as the chest CT features are similar. We believe that a SARS-CoV-2 RT-PCR should be systematically realized in case of respiratory deterioration in PAP patients.

## 1 Introduction

Pulmonary alveolar proteinosis (PAP) is a rare disease, characterized by the alveoli accumulation of surfactants proteins and lipids, hindering gas exchange [1]. Autoimmune PAP represents 90% of PAP-cases, and its diagnosis is confirmed by the presence of anti-granulocyte-macrophage colony-stimulating factor (GM-CSF) antibodies in serum. When indicated, the first-line treatment is a whole-lung lavage (WLL) [2]. Opportunistic infections - which can precede, reveal, or complicate the evolution of PAP - are a fairly common complication, and particular attention must be paid to look for them (mainly *Nocardia*, *Mycobacteria*, *Aspergillus*, *Cryptococcus* and *Histoplasma*) [3]. The severe acute respiratory syndrome coronavirus-2 (SARS-CoV-2) was first identified in December 2019, and since then progressed to a global pandemic [4]. Since then, a European retrospective cohort study reported a similar prevalence of COVID-19 in PAP-population compared to general population, but respectively higher rates of hospitalizations or death [5].

Herein, we report the case of a newly diagnosed PAP complicated by a fatal SARS-CoV-2 infection.

## 2 Case

**Fig. 1:**
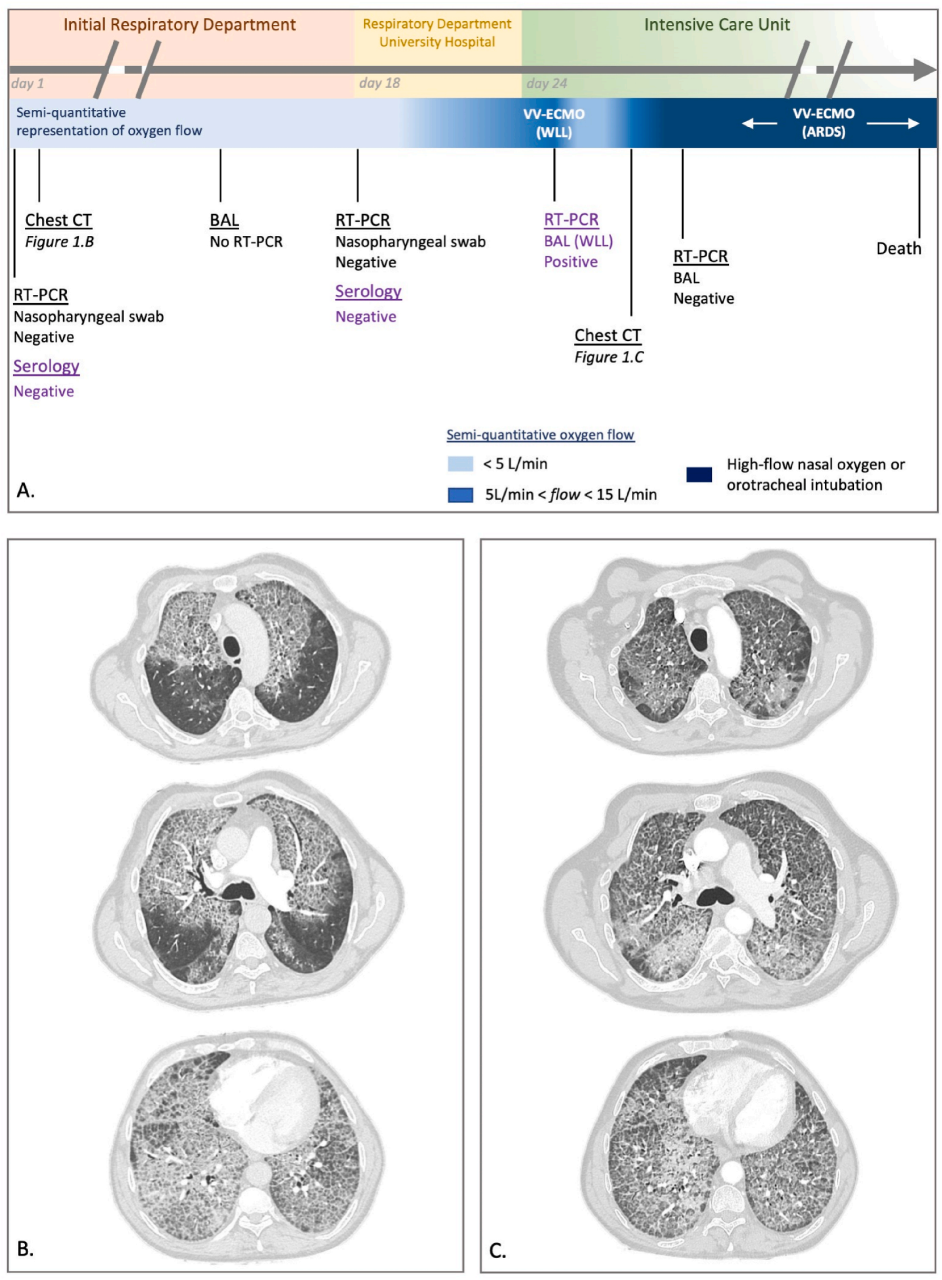
A) Chronological representation of the respiratory course and respiratory exams performed. ARDS: Acute Respiratory Distress Syndrome, BAL: bronchoalveolar lavage, CT: computed tomography, RT-PCR: reverse-transcriptase polymerase chain reaction for SARS-CoV-2, serology: specific-SARS-CoV-2 serology, VV-ECMO: veno-venous extracorporeal membrane oxygenation, WLL: whole lung lavage. Violet legend: analyses realized retrospectively (after COVID-19 diagnosis). B) First chest computed tomography (day-1). C) Second chest computed tomography (day-28). (For interpretation of the references to colour in this figure legend, the reader is referred to the Web version of this article.)

A 52-year-old man, with a past medical history of disability because of vertebral fractures following a fall, presented to a general hospital in October 2021, with a 3-months story of dyspnea. He was a farm worker, for 3 years lived in an apartment, smoked 20 cigarettes and drank 8 units of alcohol a day. He had no animal, and no previous history of allergy. He had no treatment and was not vaccinated against SARS-CoV-2. The patient reported a 10-kg weight loss in one month, a shortness breath at rest, no sputum, no chest pain, no fever, nor hemoptysis. On admission (day-1), physical examination found crackles in both lung bases, and digital clubbing. Pulse oximetry (SpO2) was 91% under 3 L of oxygen per minute and the patient had no fever. SARS-CoV-2 reverse-transcription polymerase chain reaction (RT-PCR), performed on a nasopharyngeal swab, was negative. He was hospitalized in the respiratory department to carry out an etiological assessment of this dyspnea (Fig. 1A).

Typical crazy-paving appearance was found on chest computed-tomography (CT) (Fig. 1B). The bronchoalveolar lavage (BAL) fluid had a milky aspect, did not reveal any infection (standard and prolonged bacteriological and mycological cultures), and confirmed the diagnosis of PAP by the presence of large amounts of periodic-acid-Schifft positive acellular eosinophilic lipoproteins.

The patient was then addressed to the respiratory department of a university hospital, referral centre for PAP management on November 2021 (day-18). On admission, SpO2 was 91% whereas he received 3 L per minute of oxygen. A new SARS-CoV-2 RT-PCR on nasopharyngeal swab was negative. The detection of anti-GM-CSF antibodies in serum led us to retain the diagnosis of autoimmune PAP. Investigations carried out to look for other causes were negative. The patient's clinical course was rapidly deteriorating with increasing oxygen requirement, the indication of whole lung lavage was retained, and he was transferred to the Intensive Care Unit (ICU).

At admission in the ICU (day-24), SpO2 was 93% with 12 L/min of oxygen, crackles were stable in both lung bases, and there was no fever. On day-25, the patient was intubated, before the implantation of a veno-venous extracorporeal membrane oxygenation (VV-ECMO). After the correct position control of the selective double-lumen intubation tube, a WLL was performed by instillation of warmed saline solution (14 and 15 L). The efficiency of the WLL was macroscopically good, with a progressive decrease of the BAL fluid turbidity. On day-26, VV-ECMO was removed and the patient was extubated, with SpO2 of 95% with 6 L/min.

On day-27, 48 hours following WLL, the patient presented a marked clinical worsening, with an increasing need for oxygen (up to 12L/min) and fever (39 °C). On day-28 a new chest CT was performed (Fig. 1C), showing the persistence of diffuse ground-glass opacities and the appearance of parenchyma consolidation in the basal left lung. All microbiological samples from the WLL were negative (standard and prolonged bacteriological cultures, specific search of *Nocardia* and *Actinomyces*, standard and prolonged mycological cultures, *Cryptococcus* antigen, *Aspergillus PCR*). Due to worsening respiratory condition despite high-flow nasal oxygen, the patient needed mechanical ventilation, and developed an Acute Respiratory Distress Syndrome (ARDS) [6]. A new BAL was realized on day-31 to look for a secondary infection. In the pandemic context, a third SARS-CoV-2 RT-PCR was performed, and came back positive. Retrospectively, a SARS-CoV-2 RT-PCR performed on BAL fluid removed during WLL was also positive (day-25), possibly explaining the rapid worsening of respiratory function, before the WLL. The patient was treated with dexamethasone 6 mg/d. The ARDS was managed accordingly to the guidelines [7], with the use of neuromuscular blocking agents and prone-positioning. The ventilator settings associated a tidal volume of 6 mL kg^−1^ of predicted body weight, the positive end expiratory pressure level was selected to maintain the end-inspiratory plateau pressure above 28 cmH_2_O. Despite this management, the evolution was unfavorable, and the patient was placed under VV-ECMO a second time on day-46. He finally presented an hemothorax under VV-ECMO complicated by an hemorrhagic shock, unresponsive to medical treatment, and died on day-51.

## 3 Discussion

This case report highlights the diagnostic difficulty of a COVID-19 pneumonia complicating a PAP. Indeed, they both share typical CT patterns such as bilateral and multifocal ground-glass opacities, which over time can progress towards crazy-paving areas [8]. The difficulty is that these CT patterns are classically, mimicking the features previously described in PAP [9]. In this case, the diagnosis trap was the association of a typical presentation of an auto-immune PAP (clinical history, typical chest CT images, cytologic confirmation and positive antibodies) and two successively negative SARS-CoV-2 RT-PCR on nasopharyngeal swabs (Fig. 1A). Assuming the absence of infectious disease, no molecular test has been realized on the first BAL fluid, which could have led to the earlier diagnosis of COVID-19, due to its better sensitivity than when performed on nasopharyngeal swab (93% vs 63% respectively [10]). During the second clinical worsening (2 days after the WLL, day-28), the focus was on finding an opportunistic infection, especially since PAP and opportunistic infection occur at the same time in 27% of cases [3]. Nosocomial acquisition of COVID-19 was not initially suspected for this patient, due to the absence of other current nosocomial cases in our ICU, and the lower incidence rate at that time in France [11]. The chest CT was not determining in the management of the patient because the similar features between the two pathologies, and the absence of new marked findings. In conclusion, since the emergence of COVID-19 pneumonia and due to its similar radiological patterns, we believe that a SARS-CoV-2 RT-PCR should be systematically realized in case of respiratory deterioration in PAP patients, especially in case of unfavorable evolution after WLL. To date, there is no specific feature that could allow to distinguishing these two respiratory diseases.

## Declaration of competing interest

The authors declare that they have no known competing financial interests or personal relationships that could have appeared to influence the work reported in this paper.
